# 322. Risk Factors for COVID-19 Disease Severity Using Electronic-Health Records in a Real-World Cohort in the United States

**DOI:** 10.1093/ofid/ofab466.524

**Published:** 2021-12-04

**Authors:** Shemra Rizzo, Ryan Gan, Devika Chawla, Kelly Zalocusky, Xin Chen, Yifeng Chia

**Affiliations:** Genentech, Inc., Durham, North Carolina

## Abstract

**Background:**

Over 32 million cases of COVID-19 have been reported in the US. Outcomes range from mild upper respiratory infection to hospitalization, acute respiratory failure, and death. We assessed risk factors associated with severe disease, defined as hospitalization within 21 days of diagnosis or death, using US electronic health records (EHR).

**Methods:**

Patients in the Optum de-identified COVID-19 EHR database who were diagnosed with COVID-19 in 2020 were included in the analysis. Regularized multivariable logistic regression was used to identify risk factors for severe disease. Covariates included demographics, comorbidities, history of influenza vaccination, and calendar time.

**Results:**

Of the 193,454 eligible patients, 36,043 (18.6%) were hospitalized within 21 days of COVID-19 diagnosis, and 6,397 (3.3%) died. Calendar time followed an inverse J-shaped relationship where severe disease rates rapidly declined in the first 25 weeks of the pandemic. BMI followed an asymmetric V-shaped relationship with highest rates of disease severity observed at the extremes. In the multivariable model, older age had the strongest association with disease severity (odds ratios and 95% confidence intervals of significant associations in Figure). Other risk factors were male sex, uninsured status, underweight and obese BMI, higher Charlson Comorbidity Index, and individual comorbidities including hypertension. Asthma and overweight BMI were not associated with disease severity. Blacks, Hispanics, and Asians experienced higher odds of disease severity compared to Whites.

Figure. Significant associations (odds ratio and 95% confidence intervals) with COVID-19 severity (hospitalization or death), adjusted for geographical division.

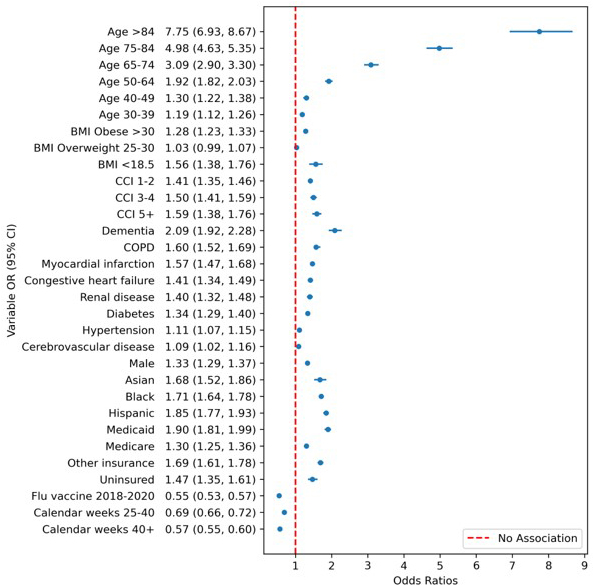

Reference and abbreviation categories: Charlson comorbidity index (CCI) = 0; Age = 18-30; Sex = Female; Race/Ethnicity = White; Insurance = Commercial; Body mass index (BMI) = 18.5-25; Calendar time = 0-25 weeks; Chronic obstructive pulmonary disease (COPD).

**Conclusion:**

Odds of hospitalization or death have decreased since the start of the pandemic, with the steepest decline observed up to mid-August, possibly reflecting changes in both testing and treatment. Older age is the most important predictor of severe COVID-19. Obese and underweight, but not overweight, BMI were associated with increased odds of disease severity when compared to normal weight. Hypertension, despite not being included in many guidelines for vaccine prioritization, is a significant risk factor. Pronounced health disparities remain across race and ethnicity after accounting for comorbidities, with minorities experiencing higher disease severity.

**Disclosures:**

**Shemra Rizzo, PhD**, **F. Hoffmann-La Roche Ltd.** (Shareholder)**Genentech, Inc.** (Employee) **Ryan Gan, PhD**, **F. Hoffmann-La Roche Ltd** (Shareholder)**Genentech, Inc.** (Employee) **Devika Chawla, PhD MSPH**, **F. Hoffmann-La Roche Ltd.** (Shareholder)**Genentech, Inc.** (Employee) **Kelly Zalocusky, PhD**, **F. Hoffmann-La Roche Ltd.** (Shareholder)**Genentech, Inc.** (Employee) **Xin Chen, PhD**, **F. Hoffmann-La Roche Ltd.** (Shareholder)**Genentech, Inc.** (Employee) **Yifeng Chia, PhD**, **F. Hoffmann-La Roche Ltd** (Shareholder)**Genentech, Inc.** (Employee)

